# Serious Game on a Smartphone for Adolescents Undergoing Hemodialysis: Development and Evaluation

**DOI:** 10.2196/17979

**Published:** 2020-09-14

**Authors:** Cristina Célia De Almeida Pereira Santana, Ana Tereza Vaz De Souza Freitas, Gilson Oliveira Barreto, Igor Sousa De Avelar, Renata Mazaro-Costa, Gina Nolêto Bueno, Diuly Caroline Ribeiro, Gabriela Damasceno Silva, Alessandra Vitorino Naghettini

**Affiliations:** 1 Hospital das Clínicas Postgraduate Program Teaching in Health Goias Federal University Goiânia Brazil; 2 Faculty of Nutrition Goias Federal University Goiânia Brazil; 3 Laboratory of Information Technology Goias Federal University Goiânia Brazil; 4 Institute of Biological Sciences Goias Federal University Goiânia Brazil; 5 Departament of Psychology Pontifical Catholic University of Goiás Goiânia Brazil; 6 Faculty of Medicine, Postgraduate Program Teaching in Health Goias Federal University Goiânia Brazil

**Keywords:** adolescent, hemodialysis, serious game, operability

## Abstract

**Background:**

Adolescents with chronic kidney disease have a hard time adhering to hemodialysis as a therapy, indicating a need to establish new alternatives for motivation and adherence to treatment.

**Objective:**

The objective of this study was to develop and evaluate a serious game to stimulate and motivate adolescents undergoing hemodialysis.

**Methods:**

We describe the technological production followed by a qualitative analysis. We invited 8 adolescents undergoing hemodialysis in the city Goiânia, located in the midwest of Brazil, to participate. The final convenience sample included 7 (87.5% of the target population) adolescents. The process was conducted in 3 phases: creation of a serious game, evaluation of its use, and observation of its motivating effect on behavioral modification with a focus on acquiring the necessary competence for self-care.

**Results:**

An app (Bim) in the modality of a serious game was developed to be used during hemodialysis; the player was encouraged to take care of a character with daily actions during his or her treatment. The game was made available to adolescents aged 10-14 years. Mobile devices were offered during the hemodialysis treatment for a period of 30-40 minutes, 3 times a week for 60 days. The usage definitions of the game were freely chosen by the participants. The qualitative evaluation of the use of the Bim app showed that it encompasses scenarios and activities that enable the exercise of daily actions for the treatment of patients. The behavioral evaluation showed that the Bim app worked as a motivating stimulus for behavioral adherence to hemodialysis requirements.

**Conclusions:**

The easy-to-access app interface showed good operability for its users. The description of the character and proposed activities contributed to motivation and ability to cope with hemodialysis care.

## Introduction

Chronic kidney disease (CKD) is a major public health problem worldwide. It is estimated that the prevalence of renal replacement therapy ranges from 18 per million to 100 per million in youth under 18 years of age [1]. In Brazil, approximately 700 young people under 19 years of age undergo hemodialysis, according to the 2017 Census by the Sociedade Brasileira de Nefrologia [2].

Hemodialysis patients are subject to extensive changes in style and quality of life due to arduous clinical and therapeutic control of the disease, in addition to recurrent hospitalizations due to complications [3,4].

The impact is greater among adolescents and negatively affects their growth and development, in addition to contributing to family disorganization, social exclusion, and the prospect of death, which simultaneously affect their families and caregivers [5,6].

With the difficulty in adhering to hemodialysis comes the need to implement new strategies to facilitate care planning and adaptation to hemodialysis treatment, in order to optimize results in self-care promotion, one of the main axes of health care for this population [5-7].

The use of games has been proposed as an effective method for behavioral changes, influencing the results [8-10] and considered as a potentially effective health intervention for treatment adherence since it motivates self-care in adolescents by the means of greater access to information on the disease [11-13].

eHealth has been reaching users of all social classes and age groups, enabling the development of tools such as serious games and digital media focused on health educational processes [8,11,13]. A study published in 2014 presented a serious game developed to stimulate care in children with hemophilia [14]; however, there have been no games in this modality aimed at adolescents undergoing hemodialysis yet [15].

Therefore, the objective of this study was to develop a serious game that would encourage adolescents undergoing hemodialysis to perform self-care and adhere to the treatment.

## Methods

### Outline

This study involved the technological production of the serious game followed by a qualitative analysis.

An app in the modality of a serious game was developed with a simulator and was complemented by a series of mini-game actions to be used by adolescents under hemodialysis; the use, motivational capacity, and game interaction of the app were evaluated.

The process was conducted in 3 phases: creation of a serious game, its evaluation while being used, and observation of the motivating effect for behavioral modification, with a focus on acquiring the necessary competence for self-care regarding the adherence to medical nutritional instructions appropriate to the condition of a dialysis patient.

### Sample

The population consisted of patients aged 10-14 years undergoing hemodialysis in the city of Goiânia, in the midwest of Brazil. The 8 patients undergoing hemodialysis in the city were personally invited by the researcher. They were all instructed about the study proposal and how it would be carried out, and an adult responsible for each participant signed a term of consent. One of the adolescents did not participate as he was transferred to another state for a kidney transplant.

The final study sample was defined by convenience and included 7 adolescents (87.5% of the target population). Participants did not need internet access to play the serious game since the app was already installed on the tablets distributed to each of them.

The exclusion criteria included refusal to participate in the study and the presence of any cognitive deficit that could possibly hinder the proper use of the app.

This study was given a favorable opinion by the Ethics Committee on Human and Animal Medical Research of the Clinical Hospital, Federal University of Goiás, under the consubstantiated opinion number 1.455.896 (CAAE: 53877316.9.0000.5078).

### Study Phases

#### Phase 1: The Creation Process of a Serious Game

The app point of origin was the idea of ​​using the Tamagotchi device that has been used by children since 1990 and bringing it to the universe of CKD. Other highlights are the applied 3-dimensional technology and visual identity of the character referring to a kidney that includes being red and having playful and easily identifiable features.

The conceptual development of the Bim app was supported by applied behavior analysis, which considers that the way individuals describe the events with which they interact changes their emotional response, which will in turn affect the behavior of either approaching (joining) those events or walking away or even fighting against them (not joining). Therefore, it is science that describes the role of individuals’ behavior [16,17].

The experiential gaming model was used to reflect on the educational design. This model proposes a sequence of elements that allow players to think over the knowledge acquired and evaluate their own performance while handling the app, which characterizes the activities as cognitive-behavioral [18].

Seeking to promote a sense of commitment in adolescents must be considered, using a contextualized and positive perspective and the capability of addressing diverse situations [19].

In the game, the graphics and icons are personalized, and immersion is favored by the interaction with scenarios, everyday situations, and caring for the character. Learning is achieved in the understanding of health needs by the means of a scoring system and a simulated response in the virtual environment.

The serious game was designed for tablets and smartphones using the Android operating system, and the Adobe Photoshop CS6, Adobe Illustrator CS6, Maya 3D, and Unity 3D programs were used. Product development was carried out at the Laboratory of Technologies and Media for Education at the Federal University of Goiás (LabTIME/UFG).

#### Phase 2: Evaluation of the Use of the Serious Game

During hemodialysis sessions, adolescents and their companions were informed about the research and invited to participate. After providing written, informed consent, tablets with the app installed were made available.

The Bim app was installed on a 7-inch Samsung Galaxy Tab Tablet model E T113, with 8GB of memory, Android 4.4 system, and Quad Core processor with 1.3 GHz. This configuration, according to developers, guarantees better operability.

Mobile devices were lent to the adolescents during hemodialysis treatment for a period of 30-40 minutes, 3 times a week for 60 days. The game usage definitions were freely chosen by the participants. Two resources were used to evaluate the use of the game: semistructured interviews and onsite observation of use.

The semistructured interviews were conducted with the users after the app availability period. We used 7 guiding questions to identify the motivation to use the app and the perception of care actions for the character. It is noteworthy that caregivers were also approached, to investigate their perceptions about the app and identify possible interfaces of use with the performance of self-care by the adolescents in the domestic or hospital environments. The interviews lasted around 20 minutes and were carried out according to the participants’ availability in the hemodialysis environment itself. They were recorded in mp4 audio and transcribed for analysis afterwards.

A weekly onsite evaluation was conducted to observe the use of the app, totaling 8 observations while the tablets were available. We selected 3 guiding criteria for observation to identify the handling of resources, interest or motivation while performing the activities, and the occurrence of favorable or opposite situations in the self-care learning process provided by the game usage.

The impressions identified in this step were recorded in a field diary.

The data obtained during the semistructured interviews and in the observation sessions were analyzed using content analysis, in the form of thematic analysis. The steps used for the data treatment were guided by Bardin [20], which allows systematizing the nuclei of meaning of the participants’ responses. The participants in this study were organized by letters and numbers, according to the order in which they were interviewed (A-adolescent).

#### Phase 3: Behavioral Evaluation

The behavioral evaluation phase of this study was conducted using applied behavior analysis methodology, which encompasses behavioral science and seeks to observe and analyze the effects of the use of the game on the behavioral pattern of the participants, focusing on the increase in adherence to the treatment demanded by the disease. In this sense, the verbal and nonverbal responses given during the use of the game by the participants in this study were taken into consideration; these responses were related to their capacity to discriminate the effects of adequate or inadequate care given to the character and its correlation to their own behavioral pattern in the face of the disease and hemodialysis treatment they underwent.

## Results

### Serious Game Creation Process

**Figure 1:**
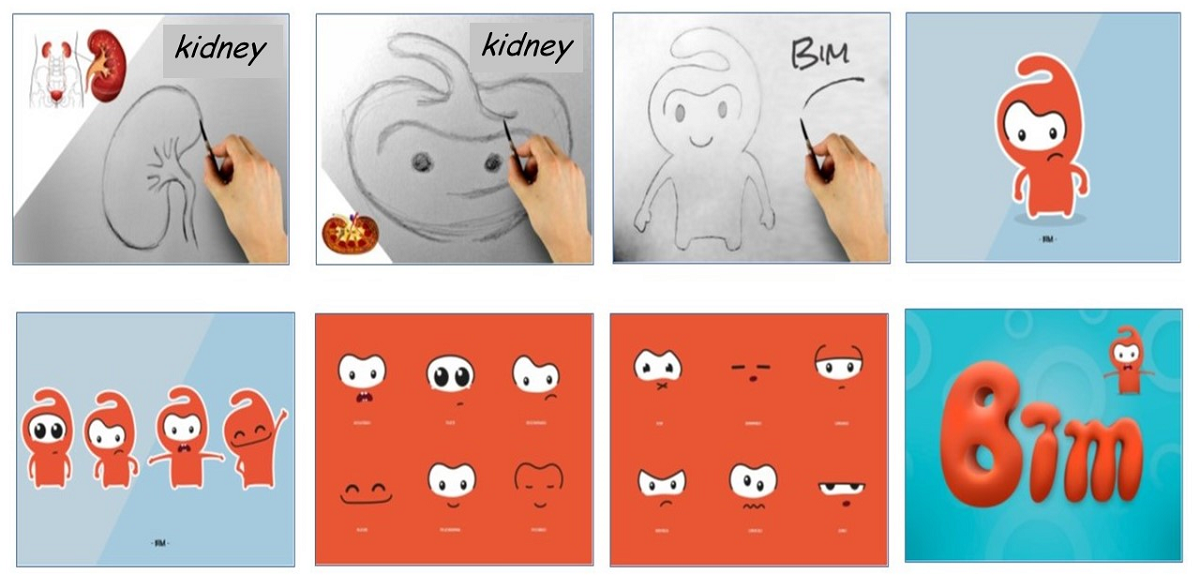
Process of creating the character, which resembles a kidney. The various expressions are meant to corroborate with the player's perception of the character's emotions while performing an action in the game.

The creation and conception process (Figure 1) required meetings with the multidisciplinary team, which included a graphic designer, game designer, nutritionist, pediatrician, nurse, biologist, educator, and psychologist. During these meetings, both the interface and all the necessary aspects of the game such as the Booleans, parameters, and environments were discussed.

**Figure 2:**
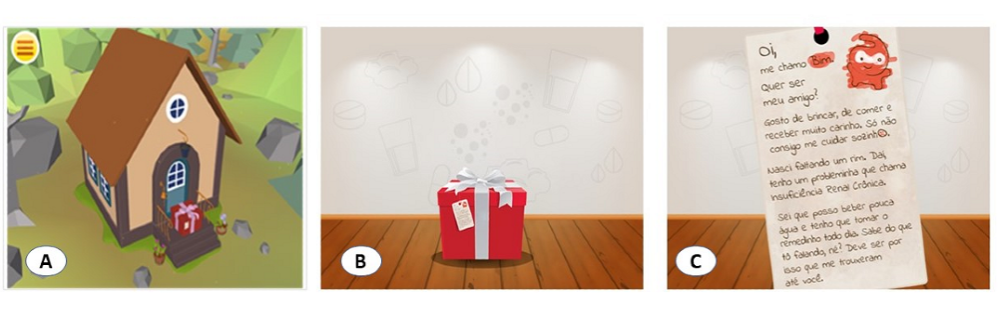
The initial app design. A: home scenario; B: gift box; C: presentation letter.

This version of the serious game offers a virtual environment that includes a home and space for dialysis treatment (Figure 2). The graphic orientation intends to stimulate task management, the ability to pay attention, and the ability to care for the character. A box with a tag (presentation letter) is used to activate it, and the tag reads (Figure 2C): “My name is Bim. Do you wanna be my friend? I like to play, eat and receive a lot of affection. I just can’t take care of myself. I have a problem called chronic kidney disease. I can drink a little water, and I need to take medicine every day. You know what I'm talking about, right? That must be why they brought me to you.”

**Figure 3:**
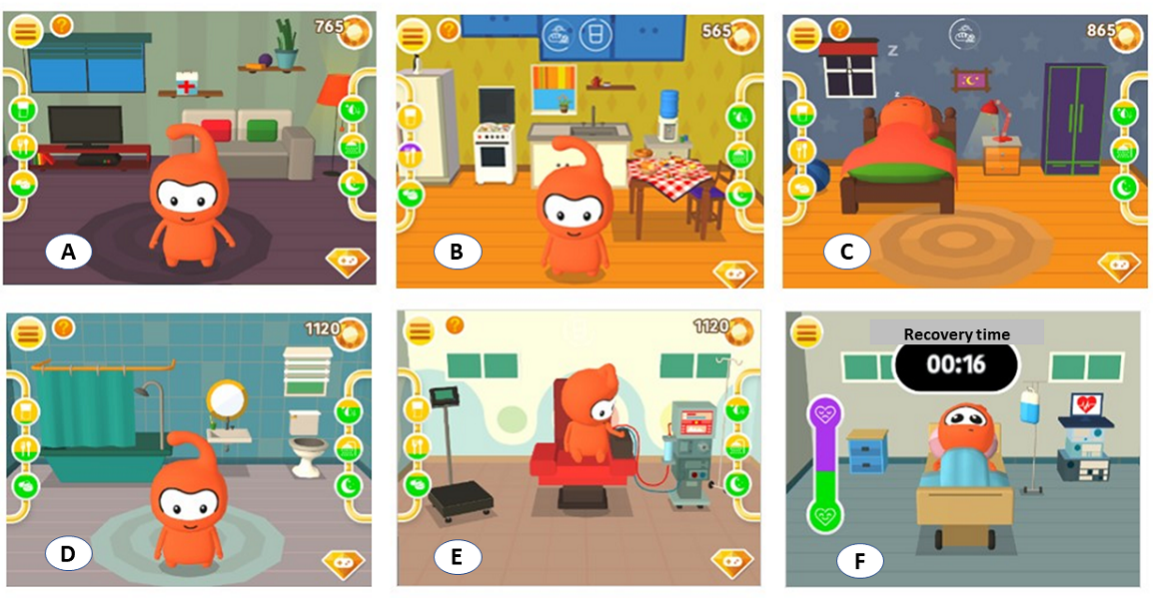
Scenarios in the serious game. A: living room; B: kitchen; C: bedroom; D: bathroom; E: hemodialysis room; F: hospital inpatient bed. At the sides, the scenarios display commands (icons and colors) that guide or indicate the proper care to guarantee a higher score.

As soon as the game starts, the adolescent is encouraged to take care of the character with daily actions that are experienced in his daily life and health condition such as: food and water intake, hygiene, use of medication, and dialysis session in several scenarios alluding to the domestic and hospital environments (Figure 3).

**Figure 4:**
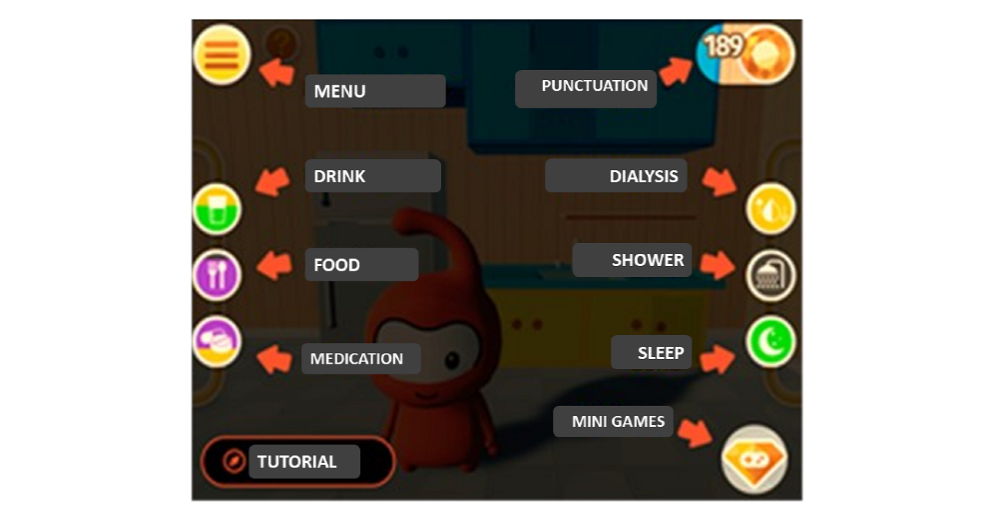
Main app interface and game parameters. Note the icons for accessing the menu, mini games, and tutorial; for viewing points; and suggesting the need for care: thirst (glass with water), hunger (fork and knife), medicine (pills), hemodialysis (blood drop and arrows), hygiene (shower), and fatigue (moon and stars). The icon colors green (normal parameter), yellow (warning sign), purple (lacking care), and black (excessive care) allow combinations where hospitalization conditions are associated with lack or excess of care and the release of points to access bonus mini games.

**Figure 5:**
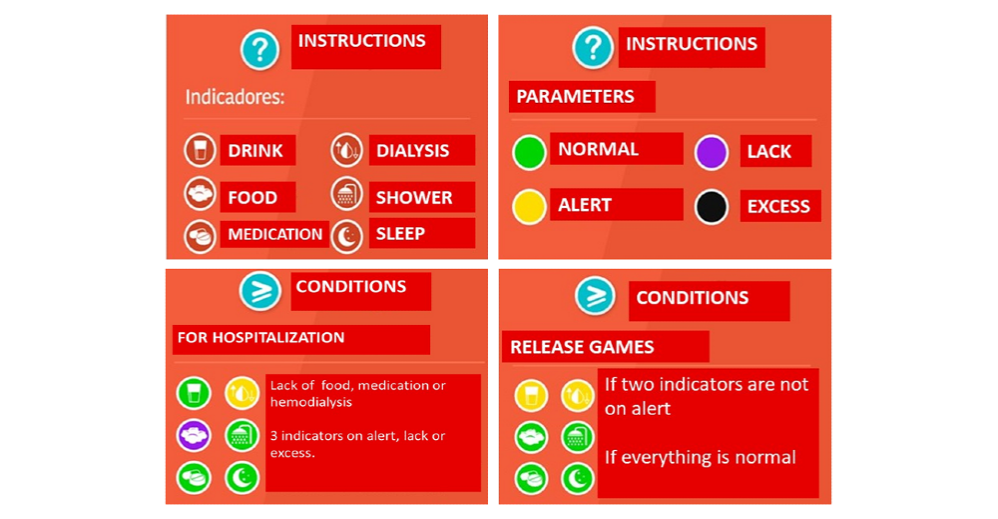
Instructions for the parameters and conditions for hospitalization and the access to bonus games.

The home screen contains self-explanatory icons. It also gives access to a tutorial that enables the user to supplement information about the use and other features of the app. The actions are guided by icons next to the screen that indicate, with colors, the need for care or its excess (Figures 4 and 5) (Multimedia Appendix 1).

The game parameters simulate the 24 hours in a day and include the following in its normal cycle: 6 meals, 3 opportunities for water intake, 4 opportunities to use medication, 1 hemodialysis session, 1 need for sleep or rest, and 2 opportunities for body hygiene. The parameters are signaled by the following colors: green (normal), yellow (alert), purple (lacking care), and black (excessive care). However, the player is free to perform a sequence of care.

**Figure 6:**
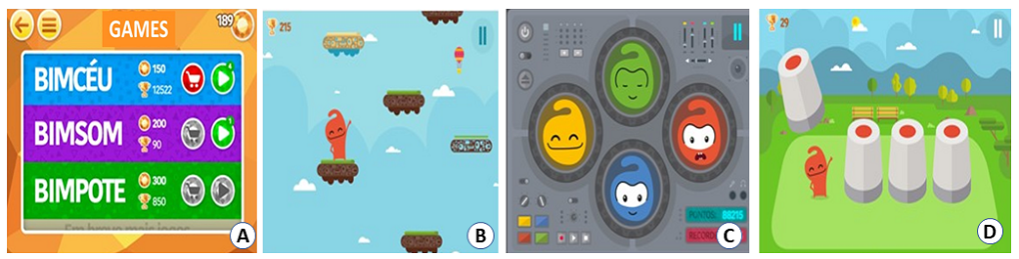
Bonus mini games. A: home screen; B: BIMCEU, a mini-game that stimulates motor coordination by ascending platforms and avoiding obstacles; C: BIMSOM, which requires the repetition of an increasingly complex sequence of sounds and colors until the final level is reached; D: BIMPOTE, which encourages memory while searching for the character.

Each activity performed by the player is mediated by a score, and each action that is considered positive or assertive is given a higher score. The positive balance of points allows the player to accumulate crystal bonuses that can be used to access mini games that stimulate motor coordination and memory. The additional gameplay provides an opportunity for more learning associated with leisure (Figure 6). Musical features and the character's interactive facial expressions are present in all the bonus games.

**Figure 7:**
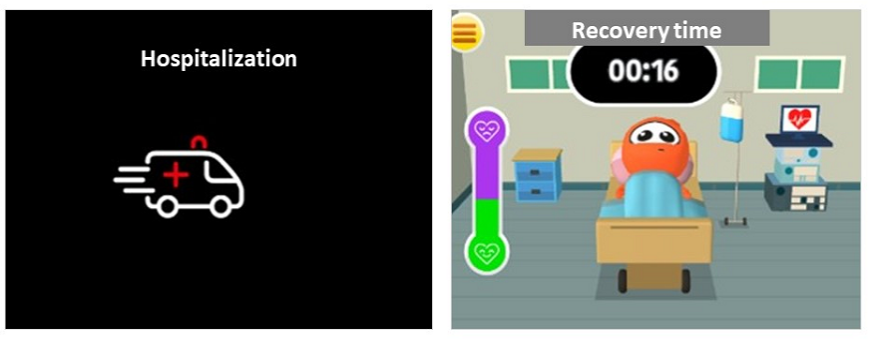
Hospitalization alert symbolized by an ambulance and the infirmary setting. The thermometer icon on the left side of the character's bed uses color to indicate the recovery and discharge time.

Situations in which the avatar, in the case of Bim, was not properly cared for could lead to its hospitalization. Once hospitalized, the character remains without access to any action for 30 seconds, as shown in Figure 7. It was expected that, during this period, the player would think about the action taken with the character, which he should have taken care of, considering the prescriptions for its health condition. Afterwards, the parameters were normalized, the game was restarted, and the accumulated bonuses were lost.

### Evaluation of the Use of the Serious Game

For this evaluation, 7 adolescents were observed, aged 10-14 years: 2 (28.6%) aged 10 years, 1 (14.3%) aged 12 years, 3 (42.8%) aged 13 years, and 1 (14.3%) aged 14 years. Of the 7 participants, 3 (42.8%) were female, and 4 (57.2%) were male. Time with CKD ranged from 6 months to 10 years; 4 (4/7, 57.2%) had been diagnosed with CKD less than 2 years ago. In the entire sample, hemodialysis treatment had started less than 3 years earlier; 5 (5/7, 71.4%) adolescents had been undergoing hemodialysis for up to 1 year. The average time of the dialysis session was 3.5-4.0 hours, 3 times a week.

In the qualitative analysis of the responses obtained from the interviews, the following categories were identified: use, motivation regarding the use of the serious game, perception of the clinical condition of the character, and care actions performed in the serious game.

The participants showed interest in the serious game, which could be verified by the interaction and motivation that led to the fulfillment of the activities proposed.

The game handling was considered simple by the participants. There was a good understanding of the main panel, easy access to scenarios, quick association of colors to the lacking of or excessive care for the character, and the perception that assertive care generated crystal bonuses and their exchange gave access to mini games (BIMCEU, BIMSOM, and BIMPOTE).

The most accessed game was BIMPOTE, and the least accessed was BIMSOM, which was quite burdensome when it came to memorization and achieving more complex levels. Table 1 shows the evaluated items and the adolescents’ impressions on the use and motivation regarding the game.

**Table 1:** Evaluation of the use and motivation to handle the Bim app.

Parameters and requirements		Response (n=7), n (%)	
		Yes	No
**Use of the game**			
	Difficulty while playing	0 (0)	7 (100)
	Good handling of the main panel	5 (71.4)	2 (28.6)
	Understanding the meaning of icons	7 (100)	0 (0)
	Understanding the meaning of colors	6 (85.7)	1 (14.3)
	I learned from the game	7 (100)	0 (0)
	Most accessed mini game: BIMPOTE	4 (57.1)	N/A^a^
	Least accessed mini game: BIMSOM	5 (71.4)	N/A
	High number of BIM hospitalizations	2 (28.6)	5 (71.4)
	Tablet crashed while playing	1 (14.3)	6 (85.7)
**Motivation**			
	I liked the character Bim	7 (100)	0 (0)
	I liked to take care of Bim	7 (100)	0 (0)
	I liked the colors and music in the game	7 (100)	0 (0)
	I would like to continue taking care of Bim	6 (85.7)	1 (14.3)

^a^N/A: not applicable.

Analysis of the content of the responses also signaled that the virtual reality proposed by the game enabled perception of the character's clinical condition, which was similar to the adolescents’ realities:

It is a good game showing our reality, of those who have kidney failure. I thought it was cool for him to undergo hemodialysis.

A1

It's [the character's routine] normal, just like mine.

A5

The game, as a playful activity, revealed potential for promoting the learning of necessary actions for self-care, providing the practice of the guidelines given by health professionals and family members:

He cannot drink a lot of water or he [ the character] might be swollen for hemodialysis.

A6

I'm already used to it ... because I know how hemodialysis is ... how my diet is.

A2

The hospitalization context appeared to be commonly associated with nonadherence to food care:

A little difficult! Because he is going to the hospital! He wants to eat something, but he can’t!

A4

Despite this, it allowed the association of unfulfilled care with the emerged complication:

I learned that if don’t take the medicine, we end up at the hospital.

A1

I learned that we have to keep a proper diet; otherwise, we’ll get sick and be hospitalized!

A7

On the other hand, it was observed that the character’s hospitalization was perceived with curiosity by the youngest participant, who repeated the nonassertive care to prove the effects of damage on the character. When she was questioned why the character was always being hospitalized, A3 replied: “I gave him a lot of water!” This adolescent was unable to adhere to water restrictions in her daily life, as reported.

### Behavioral Evaluation

The verbal reports obtained from the evaluation of the use showed that the serious game was a motivating stimulus (Sr+: positive reinforcing stimulus) not only for the patient-participants (A1, A2, A5, A7), as highlighted, but also for their caregivers and even the assisting professionals who were able to interact with a technology that taught the patients instructions they wanted them to learn and practice. The patient-participants noticed that the character’s health condition was similar to theirs; they were also able to observe the consequence of the emitted behaviors, a *sine qua non* condition for behavioral modification, which implies a wide learning process. They discriminated (Sd: discriminative stimulus) that incorrectly medicating the character (R: response to the condition of a patient with CKD undergoing hemodialysis) led to hospitalization, as well as releasing (R) a larger amount of water meant that he arrived to hemodialysis with very altered wet weight (C: aversive consequence generated by the opposite behavior to what is instructed to patients under treatment for this disease). Sensitization of the caregivers while monitoring the adolescents during the operationalization of the serious game was also observed: This made the adolescent watch the effect on the character and understand what not following the treatment instructions produced in his own organism, as highlighted by the caregiver of A4:

That’s what she goes through! She's there playing something she lives in day to day, right? She realizes what is going on in the game, nothing better, right?

caregiver of A4

A relevant description was also presented by the caregiver of A1:

Nowadays, for their age group, it’s all about game, it’s all about the internet. (...) what is really going to affect them is right there.

caregiver of A1

They learn by the means of tools, that is to say, “teaching machines,” or by the means of the serious game, “game or internet.”

## Discussion

### Principal Findings

In this study, we developed a serious game to be used by adolescents during hemodialysis sessions. This app has a straightforward interface and guidelines for game usage. Its use during the session provided the adolescent with an experience that allowed both the routine and duration of the treatment to be reduced. The activities proposed by the game also contributed to the biopsychosocial aspects and their orientation towards self-care.

Recent studies show the potential of serious games in improving young people’s health outcomes. However, few refer to its use by adolescents undergoing hemodialysis [21]. In this scenario, the individual’s interaction with virtual and multimedia resources, such as interactive games, works as a reinforcing contingency to favor learning [8,10,22-24].

The expansion and use of technology are related to overcoming mobility barriers [25]. The young population has shown good receptivity to educational electronic media, as they already use the internet and other technological resources daily [26-28].

A meta-analysis on the promotion of a healthy lifestyle by serious games has shown a positive effect; the most significant benefits were the understanding of clinical results and the maintenance of healthy long-term behaviors [29].

The insertion of serious games in both hospital and domestic contexts can lead to immersion in the experienced issue, providing an opportunity to discuss the conditions for health promotion, in addition to promoting reflection on unhealthy habits or inefficient care [14,26,28,30,31].

As far as we know, this is the first study that developed an avatar that proposed the learning of self-care focused on adolescents undergoing hemodialysis. The behavioral evaluation guided the presence of the reinforcing stimulus, which motivated the adolescents, their companions, and health professionals, and they could observe the consequence of the performed behaviors.

Games can improve the availability of reinforcing contingencies and establish motivating contexts for the modeling of necessary behavioral topographies, such as those required to accomplish the treatment of patients with CKD [21,32].

The use of serious games with playful guidance can appropriately guide the care of adolescents on hemodialysis. That said, a long-term evaluation with a larger number of patients may demonstrate interference (favoring adherence to treatment) in clinical aspects. The availability of games for caregivers can also reinforce the desired behavior change.

However, it is worth noting that even though it is an unprecedented device — a serious game for adolescents with CKD — it is still the first version and therefore needs to be optimized. In this regard, the present study opened new research horizons with the Bim app, in terms of a game, improving its navigation, improving the warnings and reminders to the user, increasing the interaction interface between the avatar and player, seeking to broaden humanization, and expanding the mini games.

When it comes to the users and caregivers, both training and engaging activities should be developed in the app; consequently, the app should be turned into part of a digital platform where both the patient and caregivers can be offered important information on the disease and how to better manage it. Expanding its perspective, the Bim app could be programed for other avatars with other chronic diseases such as obesity and cystic fibrosis.

### Conclusion

The results measured in this study emphasize the lived experience for the development and evaluation of an interactive app, in the serious game modality, to be used by patients undergoing hemodialysis. The interface proved to be a tool that contributes to the motivation and reinforcement of the care that adolescents need during treatment. Therefore, these results are expected to expand current knowledge about the effectiveness of mobile health interventions in renal replacement therapy so that this tool can be used by this population.

## Appendix

Multimedia Appendix 1 Bim app video.

## Abbreviations

CKD: chronic kidney disease
